# American Medical Association

**Published:** 1882-06

**Authors:** 


### Society Reports.

### ARTICLE III.

AMERICAN MEDICAL ASSOCIATION. Thirty-third Annual Meeting, held in St. Paul, Minnesota, June 6, 7, 8 and 9, 1882. (Special Report to the CHICAGO MEDICAL JOURNAL AND EXAMINER.)

First day, Tuesday, June 6, 11 A. M. First Vice-President P. O. Hooper, M.D., in the Chair.

After prayer had been offered by Rt. Rev. Bishop John Ireland, Dr. A. J. Stone extended the greeting of the Northwest to the delegates and members of the American Medical Association, and introduced Gov. Hubbard.

The Governor welcomed the members of the society in the name of the State. "The people of Minnesota feel highly honored to find so many distinguished physicians in their midst," said he, and in extending their greetings, he would assure the Association that if the people of Minnesota had faults, theirs was not a lack of hospitality. While he regarded the delegates' visit as highly complimentary and advantageous to the place, he believed that they would not limit their time to the fulfillment of their duties, but that they would avail themselves of the opportunity to investigate the country, note the various characteristics—as the invigorating air and regenerating climate of Minnesota—and widen the reputation of its people on their return. Said the Governor, "We invite the brain and the muscle of the world to our country." However, it was not his duty on the present occasion to show the beauty of his State; "let the meeting be the means of elevating the proud profession which you represent."

Dr. Stone then introduced some of the prominent delegates present: Drs. L. A. Sayre, of New York; N. S. Davis, of Chicago; J. M. Toner, of Washington, etc.

Several protests had been sent from various State medical societies against the action lately taken by the New York State Medical Society in revising their code of ethics. A part of these were read by the chairman, when the protest was declared unanimous. These, together with protests against a few delegates being recognized, were referred to the judicial council. Letters from the President, Dr. Woodward, now in Europe; from Dr. Sam. D. Gross, of Philadelphia, and Dr. H. D. Holton, of Brattleboro, Vt., were read, expressing their regrets that they could not be present at this meeting.

Dr. P. O. Hooper, of Little Rock, Arkansas, Vice-President of the association, and acting President of the present meeting, next read his opening address.

After a few introductory remarks, he said that the society would strive to give every one the advantage of a free hearing, and free discussion. He returned thanks to the citizens of St. Paul for their hospitality, and said that the beauty of the country had surprised every one.

A generation had now passed away since the foundation of the American Medical Association. But though most of the founders were gone forever, a few remained, and there was one among those present who had been more concerned than any other in its foundation, and without whose efforts the present meeting had never been possible.

We could now throw a retrospective glance, and look over the harmony with which the Association had been maintained. In that long period of thirty-three years, superstitions had disappeared; science had pushed its investigations in every direction. Inventions of every description had been made; so that now continents were united together. Our homes were lighted by electricity; the oceans were the channels through which flowed the commerce of the world. Moral and mental philosophy had made great strides since the time of Newton, and greater rewards had been met by modern workers. Prophets are not now without honor in their country.

New theories had been formed, and new discoveries made. The testimony of the rocks, of the sea, and of the air had been brought to confirm them, and water, steam, and electricity had been made use of in a thousand manners. All this during the life-time of the Association. The latter's purposes, however, had been to cultivate medical knowledge and progress, and in those it had kept apace with the advance of sciences. It had been liberal in its discussions, and its portals had been freely open to the worthy.

Certainly the Association had been a powerful means of promoting various sciences, but it required time, labor, and united effort to carry it out.

When the Association was founded there was no code; the medical literature of the country was in its infancy; there were few books on medicine; but since, medical societies have been founded in every State, until the American Medical Association occupied a position, prominent not only among philosophical societies, but the grandest associations in the world.

The transactions published every year had been a steady record of progress. The Association had been a specially potent factor in raising the standard of medical education, through the American Association of Medical Colleges.

A great deal had been done also in the line of scientific discoveries and achievements, which result had been appreciated in Europe as well.

A special inquisitiveness had been the characteristic of its members. Their summons had been to conquer disease, and help mankind, and in that direction had not been surpassed by any other society.

The speaker sincerely hoped that the committee in charge of the New York State Society dissention would bring the matter to an amicable settlement.

Attention to public hygiene, and to the formation of State boards of health had been the special aim of the society, which also regarded the National Board of Health as a result of their repeated efforts.

An interesting fact had attracted the Vice-President's attention, and there should be no occasion for the excessive mortality from small pox. Vaccination had been long recognized as an efficient means against that disease, and he was in favor of enforcing it on the people through the country. He was persuaded that the raging of variola was owing to neglect and ignorance on the part of some people.

There was the objection that such legislation interfered with personal rights. But personal rights were often interfered with, that is, whenever a greater benefit accrued to the community from such legislation.

Said the Vice-President: "We have, gentlemen, a Code of Medical Ethics in force in every State of the Union, and it was too late for any individual society to reject it. It was one of the results of the Association to have perfected that code, and one mistake would involve us in a world of inconsistencies, and no article should be thus tampered with.

A spirit of liberalism had always prevailed in the society, and he would not declare that no radical change would ever be inaugurated, but until such an arrangement appeared necessary to the society, we should go by the old rule. Of the twenty-three Presidents who had succeeded one another in the Association, only 13 were yet alive, and the last one, Dr. Hogden, of Philadelphia, lately deceased, had acquired a prominent place among the eminent men of his time.

"We, too, must soon yield our places to younger men," but they should know what weight will rest on their shoulders, and the Vice-President wished to be pardoned when he encouraged them to enter the field with *the zeal* of the founders of the Association. He recommended a jealous regard for the honor of the society, and a fit performance of the work fallen to their share.

A vote of thanks was returned by the members to the Vice-President, and his paper was referred for publication.

It was also resolved that the members of the Minnesota State Medical Society, then in session, should be made members of the American Association by invitation.

Then followed a reading of the names of delegates and mem bers present.

JUNE 6TH—AFTERNOON SESSION.

*Section on State Medicine and Medical Jurisprudence.* Dr. A. L. Gihon, U. S. N., chairman.

In answer to a circular from the chairman asking for information regarding medical progress, legislation, and vital statistics, reports had been forwarded from twenty two States. These reports contained a large amount of material which he had reviewed and extracted in order to present it to the members. It was laid on the table.

A communication from the Wayne County Medical Society, Indiana, was brought before the meeting. It proposed the supervision of "vaccine farms" by the National Board of Health. There seemed to have been a good deal of adulteration of the virus furnished to the trade, and death had resulted in a few cases where it was referred to the use of such impure lymph.

Dr. Haine believed that too much was asked of the general government that ought to be done by private effort. The fact that the National Board of Health would have the supervision of the vaccine farms was not a guaranty to the people that the virus would be pure. Perhaps a better plan would be to let the local Medical Societies undertake such overseeing. Or let the producers have their own supervisors, as some of them have already. Or let the producers form an association and pledge themselves to furnish reliable material. The members were divided between these resolutions, and the communication was laid on the table also.

SUICIDE IN THE CITY AND COUNTY OF PHILADELPHIA DURING THE PAST DECADE.

Dr. John G. Lee, coroner's physician of Philadelphia, read a paper with the above title. The subject had received a great deal of attention in Europe since the investigations made by Esquirol and others, but only a few authors had treated it in this country. That had been the incentive which led him to make the present researches, and he hoped that many others would accumulate statistics which would be of great value in the future.

From 1872 to 1881, inclusive, out of 12,936 cases of coroners' inquests in Philadelphia, there had been 636 suicides; 526

males and 110 females. However, these numbers were not entirely exact, for some cases of suicide must have been reported as accidental, and *vice versa,* obviously.

According to his observations, suicides increased in a locality as the population grew denser. As to color, they seemed infrequent in the black race; there being only eight cases in all. There were nearly five male to one female suicide.

The desire for self-destruction was not often manifest before the age of puberty. It was most frequent in males between the ages of thirty and forty, but in females between the ages of twenty and thirty.

In contravention to the results of European observers, he had found that suicide was not so common with the single as with the married; this the writer referred to the fact that in Philadelphia, people generally marry between the age of 20 and 25, while suicide is common at a much later age, as seen above. However, he would be guarded in his conclusions, as the material at hand was very incomplete, and he did not believe that any conclusion of real value could be formed from that. Weather records showed that a high temperature, with a low barometric pressure, were generally accompanied by an increase of self-destruction, which was also of greater frequency in the spring and the autumn. Deaths from all causes were more frequent in summer. It seemed that suicide sometimes followed a debauch, at the end of the month, after the victim had received his wages and spent them all at once; or in other cases, where many debts should have been paid. Of the number above stated, there had been 141 suicides by hanging; 122 by shooting; 96 by laudanum; 83 by cutting the throat; 57 by drowning; 27 by narcotic poisons; the rest had happened in a variety of ways. Of the 636 cases, 212 were born here; 241 in foreign countries; and 188 had not been ascertained. Hanging remained a very popular mode of death, perhaps from the supposed lack of suffering which it affords. Shooting was also a very common means in this country, where so many carry firearms. Drowning and hanging were especially frequent among the Irish, although suicides on the whole were not common among them.

Suicide, which used to be considered a crime, was not always regarded as such since the rights of man had been declared. It has never been made a crime in Philadelphia. Two hundred and twenty-eight cases had been reported as caused by insanity; generally temporary aberration. No doubt Christian charity often dictated such verdicts, but more statistics are needed in this also. The causes leading to self-destruction must be various; temperament, sociological relations, and many others. It was far from being the truth that suicides generally depended on unsoundness of the mind, except if we grant what some specialists claim, that the majority of mankind are unsound in mind. But the impulse to suicide was often impetuous, though sometimes done with a rational purpose in view, as when the victim intended to leave a life insurance to his family in need. Although there were suicides in all classes of the community, poverty was a potent factor.

The suicides themselves were often anæmic and poorly fed, so that the occurrence was in ratio to the price of food. In the writer's opinion, suicide was not on the increase, everything considered. It was rather a wonder that it was not more frequent in these days of destruction and reconstruction of societies, with the roads behind us strewed with forgotten creeds and spurious dogmas.

Motions were carried to refer the paper to the Committee on Publication.

*Section on Practical Medicine, Materia Medica, and Physiology.* G. A. Octerlony, M.D, Louisville, Chairman.

HOME TREATMENT OF PULMONARY CONSUMPTION, by general and Local Antisepsis on the Basis of Strict Individualization.

Dr. J. Hilgard Tyndale said that phthisis consisted of a destructive process in the lungs themselves, and a general septicæmia. The treatment advocated by him consisted in a local antiseptic medication of the lung, and antiseptic treatment of the general lesions, and a reinforcing of the digestion and assimilation, in order to renew the blood of the patient. He believed in changing more or less frequently the antiseptic agent used, when it seemed to lack efficiency, and on the principle that changing remedies of the same class enhances their therapeutical value.

He also altered the mode of introducing drugs, in order to prevent a local irritation of the mucous membrane, and to increase the benefit of the medication also.

THERAPEUTIC ACTION OF CHLORATE OF POTASSIUM.

Dr. John V. Schoemaker, editor of the *Medical Bulletin*, of Philadelphia, read the above paper.

This powerful and energetic drug had been discovered about the end of the last century by Berthollet, and was used with the idea that it might transmit some of its oxygen to the system. It was at first recommended as a preventive of scurvy, and Chaussier proposed its use in croup. After the remedy had almost fallen into oblivion, it was reinstated by Dr. Blanche in 1847. Dr. Shoemaker had obtained marked benefits from its internal use in scrofulous skin diseases; Dr. M. Landesberg, of Philadelphia, had also reported good results from its application to epithelioma of the eyelids. As to its physiological action, the doctor remarked that its effects seemed to be largely due to the great amount of oxygen which it contained, and it was therefore looked upon as a potent agent in the treatment and cure of all diseases in which there was sub-oxidation, or a deficiency in nutrition, secretion, excretion, aëration, and molecular change. He believed that it acted in some hitherto unexplained manner, changing the character of the blood in disease, and overcoming pathological actions. Speaking of its local application, he said that the utility of solutions of this salt used as a gargle in the treatment and cure of mercurialism was universally admitted. He used it in the proportion of one drachm to a glassful of water, as a gargle in different varieties of stomatitis. Also in inflammation and ulceration of the throat. Sometimes it seemed more efficient when used alone, at others, in combination with astringents.

Used as a gargle, or applied with a brush, or by atomization, it had been beneficial in chronic catarrh of the larynx, and of the posterior nares. In diphtheria and in phthisis he used a gargle of one to two drams to eight ounces of water; of ten grains to the ounce, in subacute or chronic otorrhœa; of ʒj to Oj, in ozæna. An injection of ʒj to a quart in leucorrhœa often proved beneficial. In gonorrhœa, gr. x to ℥j often produced an alterative action, and arrested the discharge.

It had been recommended in chronic dysentery for an injection, ʒj to ℥j. It was useful in chancroids, in solution, or dry; also in obstinate chronic ulceration, gangrenous sores, and pustular exzema. The greatest benefit experienced from its use, however, was obtained in diphtheria and croup, where it should be given in doses of from five to thirty grains three or four times daily.

In the marasmus of children small doses were useful. In anæmia, it improved the digestive tract.

In the eruptive fevers, scarlatina, measles, rotheln, and erysipelas, it brought out the eruption. It was most valuable in various diseases of the skin, ecthyma, boils, carbuncles, styes, pustular acne, eczema, sycosis. It lessens suppuration. It was also an efficient medicine in scurvy, influenza, yellow fever, rheumatism, cyanosis, hæmorrhagic diathesis, dropsy, syphilis, etc. When small doses were given they worked better before meals, large doses after.

Dr. Shoemaker generally began with small doses and increased them till its physiological action became manifest. The weak bear the largest doses. Paper was referred for publication.

*Section on Surgery and Anatomy.* Dr. W. A. Byrd, of Quincy, Ill., Chairman.

LAPAROTOMY,

By Dr. Wm. Hill, Bloomington, Ill. The chairman read this paper, the author being absent.

Dr. Hill believes that he was the first to inaugurate laparotomy. He had performed the operation in 1855 on a patient who showed obstruction of the bowels after eating unripe peaches. Anodynes, hot fomentations and cathartics had failed to relieve him, and on the third day a lump, the size of a hen's egg, appeared in the right iliac region. The case was diagnosticated an invagination of the ileum within the cœcum, necessitating surgical interference. Chloroform was administered, the peritoneum opened, and the occluding substance was pressed past the invagination. The wound was shut with hare-lip sutures, and united by first intention. A slight amount of peritonitis took place and the patient made a good recovery four weeks later. Since that time the doctor had several times met other cases of that nature where the patient died; his proposals of interference had always met a strong opposition from the profession.

DISCUSSION.

Dr. Peck, of Davenport, related a case of the same kind. The patient was on the way to recovery.

Dr. Chas. Parkes said that three difficulties arose in such cases. First, the fear on the part of the surgeon to interfere with the peritoneum; second, the difficulty of localizing the site of the trouble; third, the difficulty in ascertaining the time to make the operation. He related the case of a boy who fell on a picket fence, his bowels being made to protrude, together with the peritoneum, from the points of entrance and exit of the picket. There was no rise in temperature, no peritonitis, and the patient made a good recovery.

A paper on Anchylosis of the Hip-Joint, by Dr. Charles C. F. Gay, was read, and one on A New Truss, to be Applied after the Radical Cure of Hernia, by Dr. Jos. H. Warren, of Boston.

---

Second day—Wednesday, June 7, 1882. 10 A. M.

Report of Committee of Arrangement.

Committee on Nominations consisted of the following:

Arkansas, Dr. L. J. Dibrell; Colorado, Dr. J. Hawes; California, Dr. Orme; Connecticut, Dr. W. E. Brownson; Dakota, Dr. S. B. McGlumphy; Dist. of Columbia, Dr. W. D. Marmion; Georgia, Dr. W. F. Holt; Illinois, Dr. T. F. Worrell; Indiana, Dr. W. Lomax; Iowa, Dr. T. J. Caldwell; Kansas, Dr. J. Bell; Kentucky, Dr. L. S. McMurtry; Louisiana, Dr. J. W. Dupree; Maryland, Dr. W. Lee; Michigan, Dr. F. Pratt; Massachusetts, Dr. M. C. Parker; Minnesota, Dr. W. W. Mayo; Maine, Dr. T. A. Foster; Missouri, Dr. A. J. Steele; Mississippi, Dr. H. A. Grant; Nebraska, Dr. L. J. Abbott; North Carolina, Dr. E. Grissom; New York, Dr. N. C. Husted; New Jersey, Dr. S. S. Clark; Ohio, Dr. J. C. Scott; Pennsylvania, Dr. X. Fricke; Rhode Island, Dr. A. Callon; Tennessee, Dr. J. B. Lindsley; Texas, Dr. U. N. Park; Virginia, Dr. F. D. Cunningham; Vermont, Dr. S. W. Thayer; Wisconsin, Dr. N. Senn; U S.

Army, Dr. Perrin; U. S. Navy, Dr. Jn. M. Brown; U. S. Marine Hospital, Dr. O. W. Miller.

Dr. J. H. Packard, of Philadelphia, read the report of the committee on journalizing the transactions of the American Medical Association, of which he was the chairman.

It had been calculated that the yearly expenses of such a journal would reach $13,000 or $15,000 yearly, while the income of the association, at most, did not exceed $6,000 yearly. It was proposed that members of the association paying $5 membership fee should receive a copy of the journal, which would be forwarded to outside subscribers for $6 a year. It was also proposed that all members of local societies be made members of the association. A revision of the by-laws, a few years ago, having stated that membership should not be forfeited for non-payment of dues till the third year, some members had supposed that all they needed was to pay $5 in three years; while it was $15. It was supposed that there were 90,000 practicing physicians in this country, of whom at least 50,000 must be regular. 3,000 subscribers would be all that was necessary to carry on the new journal. It might be published by some reliable publishers, and a salaried editor. The association would need a charter, which could be obtained from any State legislature. A board of nine trustees were proposed, to be renewed, one third, every year, by the association, to take care of that matter. An editor might be appointed by the association for an unlimited time, to be replaced only by a vote of three-fourths of the members. His salary might be fixed at $6,000 a year, this including the salary of sub-editors, which he would have permission to choose. It should be published in a central place, if possible, and its title would be *The Journal of the American Medical Association.* A circular might be sent immediately to every physician in the country, in order to find out how many subscribers could be secured.

The report was accepted, and a motion passed to have copies of it distributed to the delegates the next day.

Dr. Goodwillie's motion that members present, though not delegates, have the right to vote, provoked much discussion.

Dr. Beach, of Ohio, asked the passage of the amendment by acclamation.

Dr. N. S. Davis said he did not wish to oppose the amendment if it was the desire of the majority of the delegates to pass it. He had attended all the meetings held in the past, though seldom in the quality of delegate. Every member present was expected to be a member of some local society, and, if not sent as delegate, must be represented through his society by some delegate. Would not the motion deter members from organizing and maintaining local societies, and cause the disintegration of those already existing? But the integrity and success of the American Association rested on the societies at home. Its organization had been the signal for all local societies to revive, since they were to be represented in it. The local must be in affiliation with the State, and the State with the National Association. For his own part, he had often given his privileges as delegate to some younger man. He considered that he voted through him.

The amendment was laid on the table.

Address by the chairman of Section on Practice of Medicine, Materia Medica, and Physiology. Dr. J. A. Octerlony, of Louisville, Ky.

The chairman said that labor, in medicine, had not been in a circle, but in progression. That it had advanced steadily with modern philosophy. New names had been invented to express new ideas which would not be understood by men who died twenty-five years ago. A short retrospective glance was sufficient to show what immense progress had taken place. Even in the middle of the eighteenth century measles were not discriminated from scarlet fever, Up to 1829, when Bright investigated diseases of the kidneys, nothing was known in that direction. Yet the literature he had written had only been the means to deeper researches and actual progress.

Before 1840, the very name of endocarditis was unknown. The valvular lesions, so apt to complicate acute rheumatism, had not yet been found. Exophthalmic goitre had been investigated first by Graves, of Dublin, in 1835, while it was not till 1855 that Dr. Addison discovered the pathological change which takes place in the supra-renal capsules in the disease called after him. A mere enumeration of single facts would fill up a volume.

Were one to pass in review the names of great men, it would be found that a great many had belonged to the medical profession. What the law of gravitation had done for astronomy and the law of chemical affinity for chemistry, he expected some discovery would soon happen which would do the same for medicine, in the near future. But a great deal of discrepancy existed in the profession, skepticism, which had invaded all departments of human knowledge was nowhere so marked as in medicine. It seemed strange to the human intellect that after heat, light, electricity and water had been made to serve man's desires, that he could not likewise conquer disease. But a lack of learning was not infrequent in physicians, and the number of failures among them were in ratio to their lack of knowledge. As yet we had to rely on clinical experience as our main means of curing disease. What we were able to do largely depended on what our predecessors had done; and the profession partook of the discoveries and honored a host of great unknown which had worked in its ranks. Genuine work was never lost. In reviewing the present state of medical sciences, the host of theories now brought out, though so often ephemeral, showed the activity of medical brains. Inflammation was presently regarded as almost a physiological action; a process of increased local nutrition, hastening repair. The pathology of the nervous system, so long a *terra incognita*, had now been explored, and the true nature of hyperæmia, of locomotor ataxia, and of other affections, been investigated.

It was exceedingly probably that before a long while, lesions of the brain would be as easily located as those of the heart. Phthisis was no longer regarded as an incurable disease. And its duration had been doubled under proper management, among which, pneumatic apparatuses, and especially a good training in youth, were most important. The formation of urea, it had been lately discovered, was connected with the liver, and that would throw light on enlargement and diseases of that organ. Washing the stomach was a very promising procedure. Renal pathology was also making rapid strides. Da Costa had lately traced the origin of Bright's disease to lesion of the abdominal sympathetic.

The microscope had been of special benefit in many modern discoveries. The infectious diseases by its means had been traced to the actions of parasites. Thus Eklund, of Norway, had found that lepra depended on a micrococcus; the same was true of typhoid fever. Scarlatina also depended on a parasite, while H. C. Wood had found that diphtheria had a similar origin.

Malarial diseases had been shown by Tomassi Crudeli, in 1879, to depend on a vegetable parasite, which reproduced the disease when injected into the lower animals.

Helminthology was but a recent branch of medicine. It was only in 1860 that trichinæ were found to cause epidemics of a dreadful disease, and hydatids had been investigated in 1821 for the first time. Innumerable other discoveries could never have been made without microscopes. Contrasted with these, those physicians who practiced any exclusive dogma, all irregulars, had remained in absolute inertia. Not a single original communication had ever been made by them to anatomy, physiology, pathology, gynecology, chemistry, hygiene or surgery. He expected that, in the near future, medicine would be an exact science.

### NO ADMITTANCE FOR NEW YORK DELEGATES.

In regard to the protest against the receiving of delegates from the New York State Medical Society, which was referred to us, the judicial council decide as follows:

Having carefully examined the code of ethics adopted by the New York State Medical Society at its annual meeting in February, 1882, as furnished us by the secretary of said society, the judicial council find in said code provisions essentially differing from and in conflict with the code of ethics of this association, and therefore, in accordance with provision of rule 9 of the bylaws of this association, decide unanimously that the said New York Society is not entitled to delegates in the American Medical Asssciation.

ADDRESS BY THE CHAIRMAN OF SECTION ON OBSTETRICS AND DISEASES OF WOMEN. By Dr. H. O. Marcy, of Boston.

A careful examination of uterine tumors with the microscope showed that they consisted of muscular fibers, and were properly myoma, surrounded by connective tissue of a loose texture, but never much increased. This view agreed somewhat with that of Virchow, while Billroth speaks of them in this loose manner: "These tumors appear to the naked eye as connective tissue, but might be classed with myomata." (A number of admirable microscopical preparations were thrown on a screen, which showed sections of tumors in which the connective tissue was rare, and hardly any vessels were present in the interior of the tumors. A capsule had formed around all of them, which had grown to a certain size).

Dr. Marcy declared ergot of no benefit in most of those cases. He commented on Battey's operation, and, according to the results obtained by Tait, removal of the ovaries and the Fallopian tubes was a successful treatment of uterine fibroids, but the percentage of those who succumbed to the operation was large. It was not decided whether it would not be preferable to remove the tumor itself. Hæmorrhage was a frequent cause of danger, although the means to prevent it were numerous. The doctor exhibited a rubber cloth, to be tied around the pedicle of an abdominal tumor before cutting it away, thus facilitating the operation. In opening the abdominal cavity, he always observed antiseptic precautions, and all operators were agreed in this respect, though all did not use the spray. He did not approve of draining the abdominal cavity, because the peritoneum was a large absorbing sac, only a small portion of which was injured in the operation. He believed that soon removal of the uterus would be as easy and successful as ovariotomy. But he was persuaded that antiseptic surgery was as necessary in opening into the abdominal cavity, as in other operations outside the body.

The paper was referred for publication, with the desire that the illustrations would be reproduced in the Transactions.

Wednesday Afternoon.—Section on Surgery and Anatomy.

UNUNITED FRACTURE OF THE FEMUR, Treated by Exercise. By Dr. George W. Nesbitt.

Such cases were very unfrequent, not more than one occurred in five hundred cases of fracture, so that a physician might not come across a case in a lifetime. In his case, the patient, a man twenty-nine years of age, broke the shaft of his femur after falling fifty-nine feet. He was placed under the care of an incompetent surgeon, who applied paste-board splints, and December 9, he came to Dr. Nesbitt with an ununited fracture, the limb being almost four inches shorter than the other. The general health was good. The patient in his fall had also broken both arms, which by this time had united. Attempts at reduction were made, extension and counter extension were secured, and a good deal of irritation excited. After six weeks of treatment no union had taken place. Crepitus had ceased from exudation. Feb. 2, 1878, the doctor cut open the fracture and perforated the fragments with bone drills in four places, and set the limb at rest. No union took place. Feb. 18th, iron braces were placed on the limb above and below the site of fracture, and the patient allowed to walk about, massage of the limb being practiced every day without any result. March 31 the physician drilled extensively, broke all the adhesions, and caused a good deal of inflammation to start at the site of the fracture. Nephritic colic set in, patient passed calculi, and the products of the inflammation meanwhile had been reabsorbed. July 25th, of the same year, no union having taken place yet, an elastic stocking was put on the leg, and a plaster-Paris dressing was applied, together with wire gauze, mixed, and extending the whole length of the thigh. The patient went about comfortably with this, and in November union had taken place and the patient was well.

The doctor highly recommended the use of wire gauze in connection with plaster of Paris in cases of delayed union. Following his advice, some other surgeons had tried it, and he reported several of their cases thus treated successfully. The dressing should be renewed in three or four weeks, as the muscles of the limb are liable to shrink in that period. In one case, an abscess had formed in the thigh, though the fracture had united.

DISCUSSION.

Dr. Keller, of Arkansas, called the attention of the members to the fact that these successful cases had not at first received proper treatment. That although plaster had been recommended so many times, it never took well with the profession, and a time might come when surgeons would make themselves liable to be sued for malpractice for using it, on account of the great number of cases of gangrene thus caused.

Dr. Garcelon, of Maine, related a curious case of fracture of the femur in a man, who, after a short while, was dissatisfied with his medical attendant, and resolved to prosecute him for malpractice. The patient wrapped a piece of stiff leather about his limb, hunted up testimony, and appeared in court, when all traces of a fracture were gone.

Dr. Louis A. Sayre described his mode of applying plaster-Paris dressing, and related some wonderful cases. A great deal of care and tact were necessary, besides the assistance of a competent physician. He dressed compound fractures like the simple, and after drying, he cut a fenestrum in the bandage with a knife.

A case of comminuted fracture of the humerus, with extensive external injuries, had been dressed once by him and allowed to attend to his duties on the third day. In six weeks, union was perfect. The dressings had been made to extend around the chest and the opposite shoulder, in order to fix the broken arm. He remarked that in such cases, were the patient kept in bed, the physician ought not to demand a fee, but was responsible for the patient's loss of time. He had never had a case of non-union in private practice. Plaster-Paris bandage should be applied immediately after the injury, or else after swelling had subsided, but it should not be put on a swollen limb.

He had often put plaster dressing on fractured femur, and let the patient go about on crutches in a few days, though he would recommend care in such cases. The dressing should extend around the thigh and to the opposite side of the pelvis.

Dr. McLean, of Michigan, said that from the turn of the discussion, outsiders would be led to suppose that the attending physician was always responsible in cases of non-union. That was not really the case. In spite of the most skillful treatment some cases would not unite.

Dr. McLean, now presented a patient before the assembly, a girl twenty years old, who was born with a valvus over the left eye-brow. Two pulsating tumors had grown at that point, ex tending into the orbit. He invited experienced surgeons to examine the case and advise as to the propriety of ligating the common carotid.

An operation had been made for tying the blood vessel around the tumors, six years previous, without any result. Dr. McLean advised ligature of the common carotid. Dr. Moore advised extirpation of the tumor and tying the vessels. There was likely to be much hæmorrhage but that could be controlled. Dr. E. W. Lee, of Chicago, had had experience with such cases, and believed that ligature of the common carotid was a dagerous procedure. Dr. Byrd, of Quincy, Ill., advised galvano puncture of the aneurism.

*Section on Practice of Medicine, Materia Medica and Physiology.*

TREATMENT OF SYPHILIS with subcutaneous sublimate injections. By J. V. Shoemaker, of Philadelphia.

From the introduction of the hypodermic method of medication by Alex. Wood, of Edinburg, in 1853, it had made great progress. Three years ago, Dr. Shoemaker began treating all the syphilitic patients that presented themselve to the dispensary by hypodermic injections. A glass syringe was preferable in using mercurial injections; and he liked long needles, they were not so liable to cause abscesses. He also used a needle for each patient, to prevent contagion. He began with ten minims representing $\frac{1}{8}$ grain of sublimate. In strong subjects, he increased the dose gradually, by a minim every second or third day. He diminished the dose gradually after a while. In obstinate cases, he pushed the medication till constitutional symptoms manifested themselves. In very susceptible cases he began injecting a couple of minims at first. He had obtained the best results through such treatment. The parts which he usually chose for the injection were the infra-scapular and sacral region which are the least sensitive and are also supplied with a large quantity of subcutaneous cellular tissue in which to inject the solution.

In the 113 cases treated by him, there had been neither inflamation nor abscesses. This mode of medication was especially fitted to cases of impaired digestive system; and in this case it enabled the patient to take tonics internally.

Dr. Shoemaker believed that in cases where this medication had failed, it was owing to the carelessness or lack of ability on the part of the physician.

*Section on Dentistry.* Dr. D. H. Goodwillie, of New York, Chairman.

Dr. W. C. Barrett, of New York, related a case, and exhibited casts of the teeth, showing the persistance of heredity in dental developments.

Dr. J. S. Marshall, of Syracuse, N. Y., read a paper on the need of dental surgery in the U. S. Army and Navy. Soldiers on the frontier and sailors on a long cruise had no opportunity of receiving dental services. The treatment of fractures of the lower jaw by the ordinary surgeon was now the same as it was twenty-five years ago. The inter-dental splint, invented by Dr. J. V. Bean, of Georgia, during the civil war, and improved by Dr. N. Kingsley, of New York, was a great improvement in that respect.

A committee, consisting of Drs. Allport, Marshall and Williams was appointed, to confer with the Surgeon General of the army and navy, to the effect of providing these bodies with dental surgeons.

### THIRD DAY.

*June* 8, 1882, 10 *A. M.*

Officers for the ensuing year:

President—Dr. John L. Atlee, Philadelphia.

First Vice-President—Dr. Eugene Grissom, North Carolina.

Second Vice-President—Dr. A. J. Stone, Minnesota.

Third Vice-President—Dr. J. A. Octerlony, Kentucky.

Fourth Vice-President—Dr. H. S. Orme, California.

Treasurer—R. J. Dunglison, Pennsylvania.

Librarian—William Lee, Washington.

### MEMBERS OF JUDICIAL COUNCIL.

Drs. N. S. Davis, Ill.; J. M. Brown, U. S. N.; X. C. Scott, Ohio; M. Sexton, Indiana; N. C. Husted, New York; William Lee, Maryland; J. E. Rives, West Virginia.

### OFFICERS OF THE VARIOUS SECTIONS.

Practice of Medicine.—Dr. J. H. Hollister, Ill., chairman; Dr. J. G. Lee, Penn., secretary.

Surgery and Anatomy.—Dr. W. F. Peck, Iowa, chairman; Dr. Paul F. Eve, Tennessee, secretary.

Medical Jurisprudence and State Medicine.—Dr. Foster Pratt, Mich., chairman; Dr. Thomas L. Neal, Ohio, secretary.

Ophthalmology, Otology and Laryngology.—Dr. A. W. Calhoun, Georgia, chairman; Dr. Carl Seiler, secretary.

Diseases of Children.—Dr. R. Blount, Ind., chairman; Dr. J. H. Sears, Texas, secretary.

Dentistry.—Dr. D. H. Goodwillie, New York, chairman; Dr. T. W. Brophy, Ill., secretary.

Committee on Necrology.—Dr. J. M. Toner, D. C., chairman.

Committee on Publication.—Drs. W. B. Atkinson, chairman; Thomas M. Drysdale, W. Lee, Pennsylvania; R. J. Dunglison, Albert Fricke, S. D. Gross, Casper Wistar.

Assistant Secretary.—I. N. Hines, Cleveland, Ohio. The committee announced that Cleveland, Ohio, had been selected as the place of meeting for next year.

The president appointed the following committee to nominate a board of nine trustees: Drs. L. A. Sayres, New-York; J. M. Toner, District of Columbia; J. Foster Pratt, Michigan; R. J. Dunglison, Pennsylvania; Robert Battey, Georgia; W. F. Peck, Iowa; H. O. Marcy, Massachusetts.

Dr. N. S. Davis, read the following resolutions:

*Whereas*, It appears from the amended bill making appropriations for the army for 1882–83, as recommended by the military committee of the Senate, that the amount appropriated for the support of the army medical museum and library has been reduced from $10,000 to $5,000;

*Resolved*, first, That this Association views with great regret and strong disapproval this attempt to cripple two institutions whose great value is recognized by the medical profession of the United States, as well as of Europe.

Second, That in the case of the library, whose collection of medical journals required years of unceasing effort to bring it to its present completeness, the intended reduction will be especially injurious, as from their transient character such publications would be in many instances irreplacable.

Third, That the publication now in progress of the index catalogue of the library, the most extensive work of the kind ever attempted, makes it desirable that its completeness should not be lessened by the withdrawal of means to procure current medical literature.

Fourth, That this Association express the earnest hope that Congress will restore the appropriation to its former amount, in the interests of the community at large.

These resolutions were adopted and a printed copy ordered to be sent to each member of Congress, and the heads of all the departments.

ADDRESS BY THE SECRETARY OF THE SECTION ON SURGERY AND ANATOMY—Excision of the Intestinal Canal where covered with Peritoneum, by Dr. Wm. A. Byrd, Quincy, Ill.

Successful cases of the removal of part of the intestines dated back but a few years. In most of the cases on record the patients had died. One reason was that the operation had generally been undertaken too late, after extensive adhesions had formed. The question as to the propriety of performing the operation would not be entertained now. It was too recent. But it was probable that a time would come when it would be reckoned a standard operation. Before Spencer Wells had had his success with ovariotomy, no one would call that operation a legitimate one. Resection of the digestive canal was indicated in cases of cancer, a disease which the late president Dr. Hogden, had already taught was a strictly local disease. In case of resection of the stomach, that organ should previously be washed daily with solution of salicylic acid for a few days.

Resection of part of the digestive canal was not followed by any impairment of the digestive system.

In cases of strangulated hernia with gangrene, that part of the bowel that had mortified should be removed, the two ends half brought together and sewed to the edges of the external wound, forming an artificial anus ; which, after a space of time sufficiently long should be closed by a plastic operation.

This operation consisted in dissecting the skin around the artificial anus, invaginating it so as to transform the skin into a mucous membrane of the intestine, and returning it after union, into the abdomen.

He had operated on a case October 9, 1878, for strangulated hernia, and eight inches of gangrenous intestines had to be removed. A rapid recovery had taken place and in March, 1879, he operated to close the artificial anus successfully.

In January, 1882 he operated on a case of strangulated femoral hernia in which three inches of the intestine were removed. On account of the disadvantageous situation of the hernia, Dr. Byrd made a second opening two inches above the first, and above Poupart's ligament, to the edge of which he attached the two ends of the intestine, forming an artificial anus. April 13, he operated to close the artificial anus, and the patient had made a good recovery.

### THURSDAY AFTERNOON.

*Section on Surgery and Anatomy.*

Contributions to the Surgery of the Liver; by Dr. Joseph Ranoshoff, Cincinnati, Ohio.

Operations to open abscesses of the liver were not recent achievements, but operations made in the manner to be described, had first been introduced by Dr. Marion Sims, of this country, in removing gall stones from the biliary ducts.

A precision of the diagnosis was not always necessary in order to operate. An early exploratory incision, made with ordinary care, was a legitimate procedure to make a sure diagnosis.

In dropsy of the gall bladder, aspiration of the fluid was sometimes an efficient means. But the disease was sometimes dependant on obstruction of the gall ducts from calculi.

*Case I.* Removal of calculi from the bile ducts. Patient J. R. K., a male, aged 76, had had dropsy for six months. His urine had the color of tar, he had lost fifty pounds in that time, his liver was enlarged two inches in diameter.

The gall bladder in that case was the size of the fist, hard and movable. The diagnosis was made of a gall stone, or cancer. No pain was experienced, which fact tended to exclude the latter.

The patient had suffered pain, however, five years previous. Having fixed the gall bladder by external pressure, the doctor introduced an aspiration needle which gave exit to thin bile. Passing a long needle in the direction of the bile duct, beneath the gall bladder, the needle struck a stone at a depth of four and a half inches, confirming his diagnosis; and that was, he believed, the first case on record in which a positive diagnosis of obstruction from a gall stone was made. It was decided to operate.

May third, the patient was anæsthetized, an incision four inches long, parallel to the median line was made. After the bleeding had been arrested, and the bowels, which protruded, been returned, one quart of ascitic fluid, mixed with bile, was removed, A firm concretion was now formed in the common duct, consisting of one small and two large calculi, which were removed. The omentum and intestine were cleansed with carbolized water, the gall bladder was fixed to the end of the outside incision, and morphine given.

The dressings were changed after twelve hours, after which the patient declined, and died thirty-six hours after the operation. No post-mortem was granted. One calculus weighed 138 grs., another 122.

*Case II.* A woman, æt. 32, had had a child five months previous to January 7th, 1882, during which period she lost flesh considerably. She had experienced a great deal of suffering. There were profuse night sweats; her temperature was from two to five degrees higher than normal. A smooth, round swelling, was felt in the epigastric region. An aspirating exploration was made, and January 19, 3¾ pints of pus were removed. The pain and dyspnœa diminished, and after two weeks the patient felt improved. A large quantity of pus was passed with the stools. But the abscess filled again. Feb. 22d, 1882, a solution of iodine and iodide of potassium was injected into the abscess. After ten days it had re-filled, and violent vomiting set in. A free incision was decided upon. The temperature was 105, pulse, from 120 to 150.

*Operation.*—After taking two ounces of brandy, the patient was administered ether. An incision five inches long, parallel and two inches to the right of the median line, was made into the abdominal parietes, and the surface of the liver reached; the cutting was done with the galvano-cautery, which prevented all hæmorrhage. No adhesion had taken place, and the surface of the liver was attached to the abdominal walls with wire sutures. Three and one-half quarts of pus were now removed from the abscess, which was cleansed with a five per cent. solution of carbolic acid. Iodoform was dusted over the wound. Patient recovered slowly from the influence of the ether, and was administered morphine.

March 10, she was improving. The wound was dressed and the abscess washed. A constant stream of tepid water was passed in and out of the abscess for eight hours or more a day for two weeks. Alcoholic stimulants were given. The cavity was illuminated by reflected light from a laryngoscopic mirror, and several sloughs were removed from the bottom of the cavity, which the current of water could not wash out. Œdema of the lower extremities disappeared; as also the night-sweats and diarrhœa which had been previously present. The abscess grew smaller and smaller for three months, and the patient is now quite recovered. No aspiration here had been of any avail, and Dr. Ranoshoff had seen cases die from repeated aspirations.

In the light of the discoveries of modern abdominal surgery, it was manifest that abscesses of the liver should be treated by free incision.

By means of the thermo-cautery hæmorrhage was avoided. Listerism had not been resorted to in the last operation.

The Proper Points for Incision in the Drainage of Suppurating Knee-Joints. By Dr. E. Andrews, of Chicago.

Attempts at drainage of the knee-joint often proved unsatisfactory, owing to the difficulty experienced in introducing the drainage tubes. The anatomy of that joint was not considered in the text-books in a way to help the surgeon. There were three cavities in the knee; a lower cavity covering the head of the tibia, lapping backward and downward; another cavity in front of the head of the femur, which became well-marked when distended if the knee was bent; and a cavity in the infra-patellar space. The large bursa under the quadriceps extensor, for practical purposes always present, communicated with these cavities, and an abscess sometimes extended from the knee into the thigh.

When a general suppuration of all these cavities took place, it became indispensable to anæsthetize the patient and pass drainage tubes into each one, making two openings for each cavity. One opening was to be made on each side of the bursa referred to; one on either side of the patella; on either side of the condyles of the femur, and one on either side of the ligamentum patellæ. The knee should be kept bent while making the incision. As the lower and larger cavity was almost divided in two, it would sometimes be necessary to drain both halves separately.

### DISCUSSION.

Dr. Joseph Ranashoff remarked that Dr. Byrd, in his paper on Excision of the Intestinal Canal where covered with Peritoneum, had brought out the new application of an old procedure to effect a result for which it had not been primarily intended. He converted the skin into a mucous membrane in invaginating an artificial anus, and it left the doubt in the doctor's mind, whether the skin, detached from its old blood supply, might not mortify, and endanger the life of the patient.

Dr. Prout did not recommend closure of an artificial anus. He did not think the operation justifiable, because extensive adhesions of the peritoneum had taken place.

Dr. Byrd said that in eighteen operations for strangulated hernia, he lost only one patient, and he always broke the adhesions met with. Nature could not always be trusted to. The safest plan was to be followed in such cases.

Dr. Ellis, of Michigan, related a case of artificial anus which had opened spontaneously, and the patient ultimately got well without surgical interference. A large portion of the bowel sloughed away, and the first discharge from the anus took place after two weeks.

Dr. Dora, of Illinois, said that in December last he saw the case of a woman in which a strangulated hernia had sloughed, and an artificial anus formed. No discharge had passed through the natural anus since. That illustrated the working of nature when left to itself.

*Section on Practice of Medicine,*

*Materia Medica and Physiology.* Dr. John A. Octerlony, chairman.

Salicylate of Potassium and its use in Acute Rheumatism and Dyspepsia; by Dr. M. Donnelly.

An experience of two years and a half with the use of salicylate of potassium had proved to the author its usefulness in the treatment of acute rheumatism. He had previously used alkalies in the treatment of that disease, but although neutralizing the acids and restoring the blood to its normal alkalinity, they were slow in their action. When salicylic acid was introduced the profession gave it a fair trial, but large doses of that drug were required to insure an efficient result and its use was liable to cause heart complications. Salicylate of sodium was found to be a good preparation and superseded the latter, though it did not entirely prevent pericarditis and endocarditis. The drug being a neutral salt was not enough alkaline to correct the acidity of the blood.

This induced Dr. Donnelly to look for a more alkaline salt of salicylic acid, and upon experimenting he found that two parts of bicarbonate of potassium would mix with one of salicylic acid, and form an alkaline solution. This, upon crystalizing, formed the salicylate of potassium, which is soluble in two parts of water, and is rapidly absorbed. It relieved the pain of acute rheumatism in a few hours. The urine and perspiration became alkaline after a few hours, sometimes after a few days. The metastatic character of rheumatism disappears, and the recovery is rapid.

To prevent relapse, the author gave the tartrate of iron and potassium which restored the blood to a normal condition. As to the causes of rheumatism, the profession were agreed that abnormal digestive secretions took a prominent part, the result being the formation of lactic acid in the blood.

Salicylate of potassium was most valuable in the treatment of flatulence, pyrosis and loss of appetite, and any form of dyspepsia with acidity. It was preferable to the bitter tonics in such cases.

Dr. J. A. Octerlony said that the salicylate of sodium or of potassium were especially indicated in recent and very acute cases of rheumatism accompanied with fever. In obese cases the alkalies was more efficient, in sthenic cases, aconite and veratrum viride gave the most rapid and satifactory results.. In the anæmic, he preferred large doses of the muriate tincture of iron.

### *Section on State Medicine.*

Rights of the Insane. By Dr. C. H. Hughes, St. Louis.

Dr. Hughes arrived at the following conclusions as to the rights of the insane:

I. A right to a medical inquiry, by medical men and by medical methods, into the character of their disease. They should have the benefit of a careful diagnosis by competent men accustomed to making such inquiry.

II. The right to judicial rulings on their behalf when put on trial, in accordance with medical science and not according to the judicial conception of insanity.

III. The insane should be protected from themselves and the consequences of their malady, and the State must provide asylums for them. The insane should be prohibited from marriage, for the sake of the unborn. Better not be born at all than be born insane. The State should annul all marriages with the insane so far as is consistent with public morals and the sacred marriage tie.

### FOURTH DAY.

*Friday*, June 9, 1882, 10 *A. M.*

Prayer by Rev. E. D. Neill.

*Librarian's Report.*—There had been added since the last report 167 distinct titles, exclusive of yearly volumes of transactions of societies, reports of hospitals, boards of health, and volumes of medical journals. The library consists at present of 1,702 distinct titles, or about 4,448 volumes, including pamphlets. The Boston Medical Library Association had generously placed a large number of its duplicate periodicals at the disposal of the library.

The treasurer, Dr. Richard J. Dunglison, of Philadelphia, reported a balance of $1,141.35 in the treasury. He also explained that every one attending a meeting of the Medical Association as delegate for a medical society, should thenceforth be a permanent member of the American Medical Association who should annually pay five dollars to the treasurer.

The following resolution, presented by Dr. A. L. Gihon, U. S. A. was passed:

*Resolved*, That it is the sense of the American Medical Association that it will be conducing to justice and the dignity of the profession that medical expert testimony shall be given without having the appearance of being in behalf of either side, but to be stated simply as facts.

The following resolution, by Mr. Dennison, of Colorado, was also adopted:

*Resolved*, That no action of this Association, either in its code or its annual meetings, shall be construed to commit members of the American Medical Association to the adherence of any dogma, and members should have a care not to allow their names to be erroneously registered as allopathists, etc., in State and city registration of physicians.

One thousand dollars were ordered paid the secretary, subject to the condition of the treasury.

The recommendation was made by Dr. N. S. Davis, that every other annual meeting of the Association be held in Washington.

The trustees of the proposed journal are the following:

For three years—Drs. N. S. Davis, Chicago; Moore, New York; and Toner, Washington.

For two years—Drs. Campbell, Georgia; Packard, Pennsylvania; and Connor, Michigan.

For one year—Drs. Hooper, Arkansas; Garcelon, Maine; and McMurtry, Kentucky.

Delegates to foreign societies—Drs. T. A. Emmett, D. Lewis, Wm. Carpenter and E. M. Brush, New York; and J. M. Da Costa, Pennsylvania.

Followed the address of Dr. John L. Atlee, the President-elect, and the meeting adjourned.

### SKETCHES OF THE VARIOUS OFFICERS.

JOHN LIGHT ATLEE was born in Lancaster, Pa., where he now resides, November 2, 1799. Graduated in 1820 from the medical department of the University of Pennsylvania. He was elected one of the vice-presidents of the American Medical Association in 1868; was for some time professor of anatomy in Franklin and Marshall colleges. He revived the operation of ovariotomy in 1843, and was the first to successfully remove both ovaries at one operation.

EUGENE GRISSOM, of Raleigh, N. C., was born in Granville county, May 8, 1831. Graduated from the University of Pennsylvania in 1838. Was elected to his present superintendence of the insane at Raleigh, in 1868. He holds the degree of LL.D. from Rutherford College.

JOHN A. OCTERLONY is a resident of Louisville, Ky. Born in Sweden in 1838, he graduated from the University of New York in 1861. Has been lecturer on chemical medicine in the University of Louisville, and is now professor of practice in the Kentucky School of Medicine.

ALEXANDER J. STONE. Born in 1845, at Wiscasset, Me. Graduated from the Berkshire Medical College, at Pittsfield, Mass., in 1868. Settled in St. Paul in 1870. Founder of the St. Paul Medical College, he holds the chair of Diseases of Women in that institution, at present known as the Minneapolis College Hospital.

HENRY S. ORME, of Los Angeles, Cal., was born in Milledgville, Ga., 1837. Graduated from Oglethorpe University in 1858, and from the New York University in 1861.

R. J. DUNGLISON. Born in 1834. Graduated from Jefferson College. Edited Dunglison's Medical Dictionary in 1874. Has been assistant secretary of the International Medical Congress.

J. M. TONER, of Washington, was born in Pittsburgh, in 1825, and graduated from Jefferson Medical College in 1853; author of the "Toner Lectures."

N. S. DAVIS, LL.D., of Chicago, graduated from the College of Physicians and Surgeons of the Western District of New York, in 1837; is editor of the CHICAGO MEDICAL JOURNAL AND EXAMINER, professor of Practice in the Chicago Medical College, Fellow of the Chicago Academy of Sciences, and is widely known through his medical writings.

W. B. ATKINSON, of Philadelphia, was born in Haverford, Pa., 1832. Graduated from Jefferson Medical College in 1853, he became Permanent Secretary in 1864. Well-known as the author of a Biographical Dictionary of American Physicians and Surgeons.

ISAAC N. HINES, of Cleveland, O., was born at Shippensburg, Pa., 1834. Graduated from the New York Medical College of Physicians and Surgeons. He is professor of physiology and pathological histology in Cleveland Medical College.

